# Genetic mutations in NF-κB pathway genes were associated with the protection from hepatitis C virus infection among Chinese Han population

**DOI:** 10.1038/s41598-019-47058-y

**Published:** 2019-07-25

**Authors:** Ming Yue, Ting Tian, Chunhui Wang, Haozhi Fan, Jingjing Wu, Jinke Wang, Jun Li, Xueshan Xia, Amei Zhang, Rongbin Yu, Yun Zhang, Peng Huang

**Affiliations:** 1 Department of Infectious Diseases, The First Affiliated Hospital of Nanjing Medical University, Nanjing, 210029 China; 2 Department of Acute Infectious Disease Control and Prevention, Jiangsu Province Center for Disease Prevention and Control, Nanjing, 210009 China; 3 Institute of Epidemiology and Microbiology, Eastern Theater Command Centers for Disease Control and Prevention, Nanjing, 210002 China; 4 Department of Epidemiology and Biostatistics, Key Laboratory of Infectious Diseases, School of Public Health, Nanjing Medical University, Nanjing, 211166 China; 5 State Key Laboratory of Bioelectronics, Southeast University, Nanjing, 210096 China; 6 Faculty of Life Science and Technology, Kunming University of Science and Technology, Kunming, 650500 China

**Keywords:** Genetic association study, Viral infection, Genetic association study, Viral infection

## Abstract

Host genetic polymorphism is one of major unalterable major factors for HCV infection. NF-κB proteins play multiple roles in immune response and involve in HCV infection and progression. This study was conducted to explore the relationship between single nucleotide polymorphisms (SNPs) in NF-κB pathway and the susceptibility as well as resolution of HCV infection. A total of 1642 Chinese subjects were enrolled in the study, including 963 uninfected control cases, 231 cases with spontaneous viral clearance and 448 cases with persistent HCV infection, and four SNPs (Rel rs842647, NF-κB2 rs12769316, RelA rs7101916, RelB rs28372683) were genotyped by TaqMan assay among them. Potentially functional polymorphisms were analyzed using online bioinformatics tools. The logistic analyses results indicated that RelA rs7101916 T allele (*P*_*Bonferroni*_ = 0.016) and RelB rs28372683 A allele (*P*_*Bonferroni*_ = 4.8e-5) were associated with an decreased risk of the susceptibility to HCV infection among Chinese Han population, which were consistent with the results of cumulative effects and haplotype analysis. The *silico* analysis of SNPs function suggested that the genetic variation of rs7101916 and rs28372683 could influence gene transcriptional regulation and expression, subsequently affecting NF-κB pathway activation and the susceptibility to HCV infection. This study firstly reported that the carriage of RelA rs7101916 T or RelB rs28372683 A was the potential protective factor against HCV infection among the Chinese population.

## Introduction

Over 185 million people worldwide suffer from hepatitis C virus (HCV) infection^1,2^. HCV infection is usually asymptomatic and will develop some hepatic and extrahepatic disorders, including liver cirrhosis, hepatocellular carcinoma, non-Hodgkin’s lymphoma, cryoglobulinemia, etc^1,2^. In recent years, due to the highly effectiveness of clinical applications of novel direct-acting antiviral drugs (DAAs), the expectation for a cure for hepatitis C has increased dramatically^3^. However, many significant challenges remain, including undetected most HCV cases, resistance-associated variants (RAVs) of HCV protease inhibitors, and HCV reinfection after cure of chronic hepatitis C, etc^4^. A critical measure in the global control of hepatitis C is identifying atrisk persons for HCV^5^.

Host genetic polymorphism is one of unalterable major risk factors for HCV infection. Genome-wide association study (GWAS) found that single nucleotide polymorphisms (SNPs) on the *IL28B* gene are associated with HCV clearance^6^. In addition, various human leukocyte antigen (HLA) alleles involved in the immune response have been shown to be associated with spontaneous clearance of HCV infection, and may even predict HCV treatment response^7^. Nuclear factor κB (NF-κB) was first discovered as a nuclear factor binding to a κ enhancer of the immunoglobulin κ light chain gene of activated B cells^8^. NF-κB can be found in almost all animal cell types and is a key an important regulator of the anti-pathogen immune response, inflammatory reaction, cell proliferation and survival^9,10^. It subsequently became clear that NF-κB is involved in signal-induced expression of hundreds of genes^9,10^. The NF-κB transcription factor family is composed of five DNA binding proteins: NF-κB1 (p50, p105), NF-κB2 (p52, p100), RelA (p65), RelB, and c-Rel^11^. These proteins can form homodimers or heterodimer with widely different transcriptional activities and their expression levels show differences in event and tissue-specific expression patterns in response to a different stimulus^11^. NF-κB remains in an inactive form complexes with inhibitors of κB (IκBs), of which mainly includes IκBα, IκBβ, and IκBε^12^. Different IκBs control the regulation of different tissues by inhibiting specific NF-κB dimers^12^. These inhibitory proteins can be phosphorescent and degraded by IκB kinase (IKK) in a critical event leading to NF-κB activation^13^.

A variety of external stimulants can activate NF-κB signaling pathway, including viruses, bacterial lipopolysaccharide, proinflammatory cytokines and stress-inducing agents, followed by the promotion of the expression of hundreds of genes. Most of target genes encoded proteins, including many cytokines, chemokines and receptors, participate in the process of immune recognition and adhesion, antigen presentation in the host innate and adaptive immune response^9^. NF-κB also can be activated by HCV and stimulates the production of IL-1, IL-6, lymphotoxin, and IFN-γ^14^. Due to the extensive influence, NF-κB can be regarded as the central mediator of the immune response^9,15^. The immune response to HCV is mainly regulated by NF-κB and interferon-signaling pathways^9,15^. Therefore, abnormal activation or regulation of NF-κB signaling pathway genes are associated with various immune-related diseases, including some infectious diseases, autoimmune diseases, chronic obstructive pulmonary disease, and cancer^9,10,16–18^.

Many SNPs in the NF-κB signaling pathway genes have been identified involved in many diseases. Our previous studies found that the nearby RelA rs11820062-A and NFKB1 rs28362491-D are significantly associated with an increased HCV susceptibility within a high-risk Chinese population and they may alter the binding of transcription factor (TF) and transcriptional regulation of the corresponding gene^19,20^. In addition, in the previous bioinformatics analyses, rs11820086 also may be related to RelA mRNA expression in immortalized B-lymphocytes^21^ and conferred increased susceptibility to chronic kidney disease^22^ and schizophrenia^21^. Other researches have suggested NF-κB1 rs230530, NF-κB2 rs1056890 and other SNPs are linked to many diseases, such as liver cancer^23^, alcohol addiction^24^, the inflammatory responses in the development of lymphedema following breast cancer treatment^25^, the development of multiple myeloma^26^ and other immune-related diseases^27,28^. No genetic association studies of rs7101916 was reported.

Considering the role of NF-κB signaling pathway genes in the innate and adaptive immune response to HCV infection, our previous studies deserve continuing, in-depth investigation. Therefore, this case-control study aims to further explore the relationships between NF-κB pathway genes’ SNPs: Rel rs842647, RelA rs7101916, NF-κB2 rs12769316 and RelB rs28372683, and HCV infection outcomes within a high-risk Chinese population.

## Materials and Methods

### Ethics statement

This study was given permission by the institutional review board of Nanjing Medical University and was implemented in accordance with the ethical guidelines of the Declaration of Helsinki^29^. Written informed consent was collected from all participants before entering into the study.

### Study population

**Figure 1:**
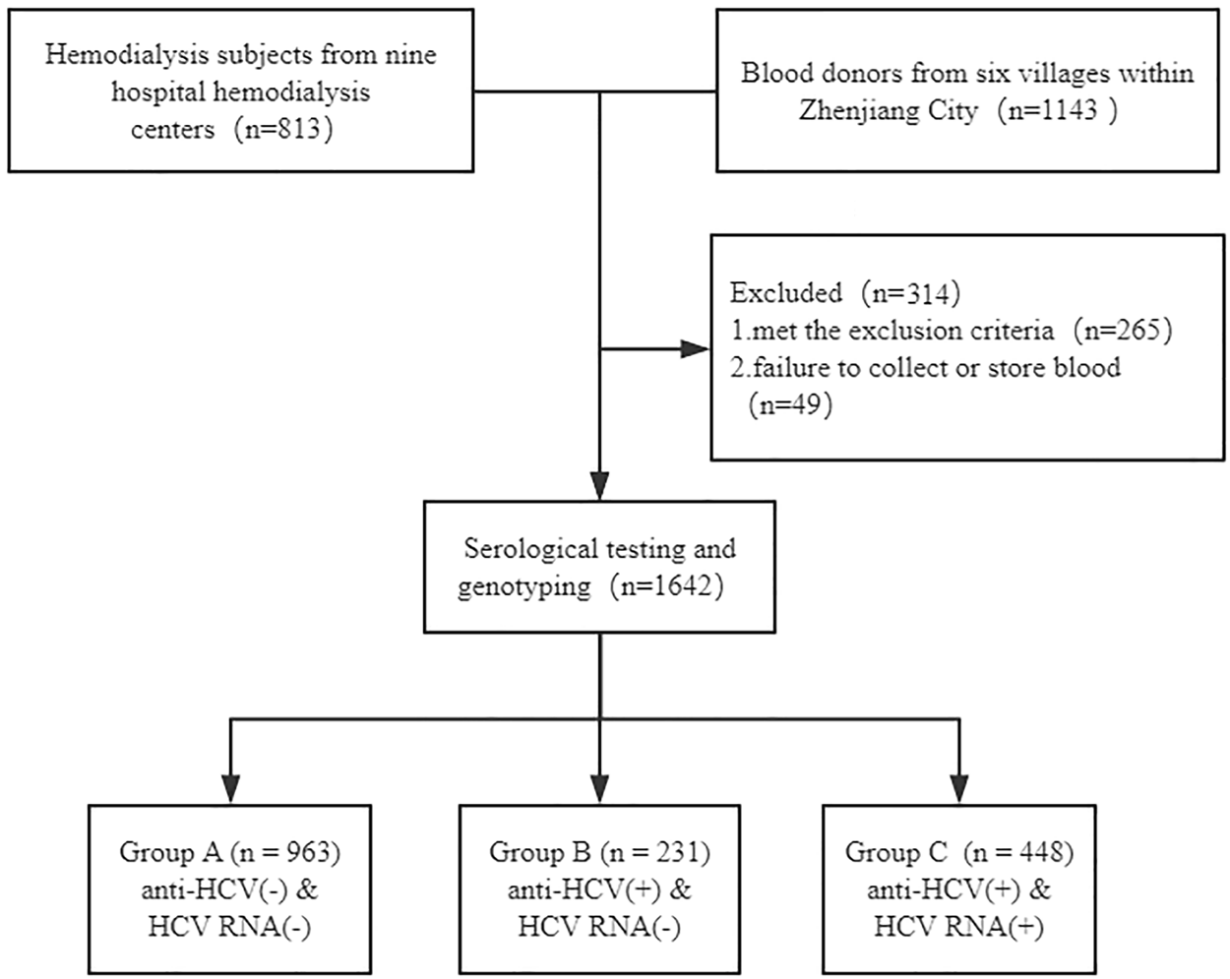
Flowchart of the selection of patients included in the study.

This study conducted a case-control study with a total of 1642 subjects, including 693 hemodialysis (HD) subjects recruited from nine hospital hemodialysis centers in southern China from October 2008 to May 2015, and 949 paid blood donors recruited from six villages within Zhenjiang City from October 2008 to September 2016. Subjects co-infected with hepatitis B virus (HBV) or human immunodeficiency virus (HIV), or suffered from autoimmune, alcoholic or metabolic liver diseases, or received polyethylene glycol (Peg) IFN-α plus ribavirin (RBV), or oral DAAs treatment were excluded from this study. The flowchart of the selection of patients included in the study was shown in Fig. 1.

The subjects were put into three groups depending on their anti-HCV and HCV RNA results. Group A was comprised of HCV-uninfected controls with seronegative anti-HCV and seronegative HCV RNA. Group B was composed of spontaneous clearance subjects with seropositive anti-HCV and seronegative for HCV RNA. Group C was composed of persistent infection patients with seropositive anti-HCV and seropositive HCV RNA. Individuals in group B or group C were defined as infected individuals. All results of serologic tests were verified by three separate experiments within the 12 consecutive months. The control subjects (group A) were matched to the infected individuals (group B or group C) by age (5-year interval), gender and the village of recruitment.

Structured questionnaires were administered to trained interviewers for interviews carried out with every participant to collect the demographic information, the environmental exposure history and the medical history of HCV infection. Quality control program was established to guarantee the reliability of all data obtained.

### Viral testing

Venous blood samples were drawn from each participant after the interview and stored at −80 °C after centrifugation until the experiment. Anti-HCV antibodies were detected using a third-generation enzyme-linked immunosorbent assay (ELISA: Diagnostic Kit for Antibody to HCV 3.0 ELISA, Intec Products Inc, Xiamen, China) according to the manufacturer’s instructions. HCV RNA was extracted using Trizol LS reagent (Takara Biotech, Tokyo, Japan), and reverse transcription polymerase chain reaction (PCR; Takara Biotech) was performed. The Murex HCV Serotyping 1–6 Assay ELISA kit (Abbott, Wiesbaden, Germany) was used to detect type-specific antibodies of various HCV genotypes^30^.

### SNPs selection

TagSNP (Rel rs842647) was selected using Haploview software (version 4.2; Broad Institute, Cambridge, MA, USA) on the basis of the data of HapMap Phase II CHB (Chinese in Beijing) obtained from 1000 Genomes Project resources (http://www.1000genomes.org/). RelA rs7101916, NF-κB2 rs12769316 and RelB rs28372683 were selected based on the functional prediction for possible transcription factor binding sites or miRNA binding sites using National Institute of Environmental Health Sciences (NIEHS) (https://www.niehs.nih.gov/), Search RegulomeDB (http://www.regulomedb.org/) and based on the related literatures in which SNPs were reported to be associated with immune-related diseases. In addition to the above strategies, the minor allele frequency (MAF) of the candidate SNP must be more than 5% among the Chinese Han population.

### Genotyping assays

Genomic DNA was extracted from leukocytes derived from subjects’ blood samples using protease K digestion, phenol-chloroform extraction and ethanol precipitation. Before the genotyping, we used ultraviolet spectrophotometer (UV-2700220V CH) to detect the concentration and purity of DNA, and excluded OD_260_/OD_280_ < 1.75, OD < 100 ng/ul sample. Candidate SNPs were genotyped by using the TaqMan allelic discrimination assay on an ABI 7900HT Real-Time PCR System (Applied Biosystems, Foster City, CA, USA). The primers and probe sequences for candidate SNPs are shown in Supplementary Table 1. The experimenters were blind to the subjects’ demographical and clinical data. Quality control was performed using two negative controls and two positive controls included in each 384-well plate to identify the agent or system contaminations. The success rates of genotyping for four SNPs were all above 99.8% and a 100% concordance rate was showed in 10% random selected samples for repeated testing. The genotyping results were analyzed using Sequence Detection System software (SDS, version 2.3; Applied Biosystems, Foster, CA, USA).

### *In silico* analysis

SNPInfo web server (http://snpinfo.niehs.nih.gov/) was run based on the data from HapMap China Han Beijing (CHB) population to predict the impact on genes of potentially functional polymorphisms. The Vienna RNAfold web server (http://rna.tbi.univie.ac.at/cgi-bin/RNAWebSuite/RNAfold.cgi) was used to predict secondary structures of single stranded RNA sequences based on the latest ViennaRNA Package (Version 2.4.4). The secondary structure with the minimum free energy (MFE; i.e. MFE structure) or the minimal base pair distance (i.e. centroid secondary structure) was computed and drew to characterize and compare the the influence on the secondary structure of gene between wild type and mutant type of the polymorphism site. We also carried out the expression Quantitative Trait Loci (eQTL) analysis on the public database Genotype-Tissue Expression (GTEx) Portal (http://www.gtexportal.org/home) to identify whether this SNP could cause any differential expression in different tissues.

### Statistical analysis

All data were analyzed using Statistical Package for the Social Sciences software (SPSS: version 21.0.0.0; SPSS Institute, Chicago, IL, USA) and Statistical Analysis System software (SAS: version 9.4; SAS Institute, Cary, NC, USA). Demographics data, clinical characteristics and genotype distribution of subjects were compared using one-way analysis of variance (ANOVA), χ^2^-test or Kruskal-Wallis test where appropriate. A goodness-of-fit χ^2^ test was used to estimate Hardy-Weinberg equilibrium (HWE) for each SNP among the controls. Linkage disequilibrium (LD) parameters (r^2^ and D′) were calculated using Haploview software (version 4.2; Broad Institute, Cambridge, MA, USA). The associations between SNPs with the susceptibility to HCV infection or the spontaneous clearance of HCV infection were estimated by comparing group A v.s. group (B + C) or group B v.s. group C, respectively, and were calculated by binary logistic regression analysis, unadjusted or adjusted for age, gender and route of infection, and were explained by odds ratios (ORs), 95% confidence intervals (CIs) using dominant genetic model ((homozygous type + hybrid type) v.s. wild type). Cumulative effects of combined risk alleles or combined risk genotypes were estimated with logistic regression model, adjusted for gender, age and routes of infection or calculated by the Cochran-Armitage trend test. Haplotype reconstruction was carried out using the PHASE software (version 2.1; UW TechTransfer Digital Ventures, University of Washington, Seattle, WA, USA). *P* <0.05 in a two-sided test was considered statistically significant. Bonferroni correction was utilized to conduct multiple comparisons.

## Results

### Basic characteristics

The demographics and clinical characteristics of all subjects are presented in Table 1. No significant age or gender difference was found among three groups (all *P* > 0.05) or between any two groups (all *P* > 0.05, data not shown). However, the alanine aminotransferase (ALT) levels, aspartate aminotransferase (AST) levels, routes of infection and HCV genotype were significantly different among three groups (all *P* < 0.01) or between any two groups (all *P* < 0.01, data not shown). The allele distribution of four SNPs was in accordance with Hardy-Weinberg equilibrium expectations in the control group (group A: *P* = 0.120 for rs842647, *P* = 0.271 for rs7101916, *P* = 0.153 for rs12769316, *P* = 0.603 for rs28372683).

**Table 1:** Demographical and clinical characteristics among HCV control, spontaneous clearance and persistent infection groups.

Variables	Group A (n = 963)	Group B (n = 231)	Group C (n = 448)	*P* value
	n (%)	n (%)	n (%)	
Age (years, mean ± SD)	55.08 ± 11.29	56.63 ± 9.26	54.74 ± 8.72	0.068^a^
Gender				0.694^b^
Male	357 (37.1)	80 (34.6)	158 (35.3)	
Female	606 (62.9)	151 (65.4)	290 (64.7)	
ALT(median(IQR),U/L)	10.00 (6.00, 18.00)	23.00 (11.00, 40.00)	28.00 (17.00, 50.00)	<0.001^c^
AST (median(IQR), U/L)	18.00 (11.00, 24.75)	28.00 (17.00, 40.00)	34.50 (24.00.50.75)	<0.001^c^
Routes of infection				<0.001^b^
HD	547 (56.8)	84 (36.4)	62 (13.8)	
Blood donation	416 (43.2)	147 (63.6)	386 (86.2)	
HCV genotype				<0.001^b^
1	—	40 (45.5)	93 (36.0)	
Non-1	—	27 (30.7)	11 (4.3)	
Mixed	—	21 (23.9)	154 (59.7)	

Abbreviations: SD: standard deviation; ALT, alanine transaminase; AST, aspartate aminotransferase; ANOVA, analysis of variance; HCV, hepatitis C virus; HD, hemodialysis.

Group A: controls; Group B: spontaneous clearance subjects; Group C: persistent infection patients.

^a^*P* value of one-way ANOVA among three groups.

^b^*P* value of χ^2^-test among three/two groups.

^c^*P* value of Kruskal–Wallis test among three/two groups.

### Association between *NF-κB* genes polymorphisms and the susceptibility to HCV infection

Genotype distribution of SNPs rs842647, rs7101916, rs12769316 and rs28372683 among three groups is shown in Table 2. It can be seen that the allelic and genotypic frequencies of rs7101916 and rs28372683 were significantly different across the three groups (*P* < 0.05). However, no significant difference was seen in the distribution of rs842647 and rs12769316 genotypes among the three groups. Only genetic dominant model was used for analyze the association between SNPs and the susceptibility to HCV infection, because the number of the subjects carried the minor allele homozygote of two SNPs (rs842647 and rs12769316) among some groups was small (<10) for powerful statistical analysis. As shown in Table 3, before or after adjusting for gender, age, and the route of infection, the results of logistic regression analysis showed that, compared with the major allele homozygote (rs7101916 CC genotype or rs28372683 CC genotype), the carriage of rs7101916 T allele or rs28372683A allele was associated with a decreased risk of the susceptibility to HCV (rs7101916: OR = 0.728, 95% CI = 0.588–0.901, *P* = 0.004; rs28372683: OR = 0.499, 95% CI = 0.366–0.681, *P* = 1.2e-5), and they remained significant after the multiple comparisons using Bonferroni correction (rs7101916: *P* = 0.016; rs28372683: *P* = 4.8e-5).

**Table 2:** Genotypes distributions of *NF-κB* signaling pathway genes among persistent infection, spontaneous clearance and control group.

SNPs	Genotypes	Group A, n (%)	Group B, n (%)	Group C, n (%)	*P*
rs842647	GG	674 (70.0)	162 (70.1)	310 (69.2)	0.648
	GA	271 (28.1)	61 (26.4)	127 (28.3)	
	AA	18 (1.9)	8 (3.5)	11 (2.5)	
	G allele	1619 (84.1)	385 (83.3)	747 (83.4)	0.865
	A allele	307 (15.9)	77 (16.7)	149 (16.6)	
rs7101916	CC	372 (38.6)	108 (46.8)	219 (48.9)	**0.003**
	CT	440 (45.7)	88 (38.1)	176 (39.3)	
	TT	151 (15.7)	35 (15.2)	53 (11.8)	
	C allele	1184 (61.5)	304 (65.8)	614 (68.5)	**0.001**
	T allele	742 (38.5)	158 (34.2)	282 (31.5)	
rs12769316	CC	679 (70.5)	157 (68.0)	295 (65.8)	0.335
	CT	252 (26.2)	65 (28.1)	130 (290)	
	TT	32 (3.3)	9 (3.9)	23 (5.1)	
	C allele	1610 (83.6)	379 (82.0)	720 (80.4)	0.105
	T allele	316 (16.4)	83 (18.0)	176 (19.6)	
rs28372683	CC	799 (83.1)	204 (88.3)	399 (89.1)	**0.001**
	CA	154 (16.0)	21 (9.1)	47 (10.5)	
	AA	9 (0.9)	6 (2.6)	2 (0.4)	
	C allele	1752 (91.1)	429 (92.9)	845 (94.3)	**0.010**
	A allele	172 (8.9)	33 (7.1)	51 (5.7)	

Group A: controls; Group B: spontaneous clearance subjects; Group C: persistent infection patients.

**Table 3:** Association between SNP in *NF-κB* signaling pathway genes and the outcomes of HCV infection.

SNPs	Genotypes	OR (95% CI)^a^	P^a^	OR(95% CI)^b^	P^b^/P^c^
**(a) SNPs associated with the susceptibility of HCV infection, Group (B + C)** ***vs*** *.* **Group A**					
rs842647	GG	—	—	—	—
	GA/AA	1.023 (0.826–1.267)	0.836	0.887 (0.705–1.117)	0.308/1.232
rs7101916	CC	—	—	—	—
	CT/TT	**0.678 (0.555–0.827)**	**1.23e-4**	**0.728 (0.588–0.901)**	**0.004/0.016**
rs12769316	CC	—	—	—	—
	CT/TT	1.201 (0.972–1.483)	0.090	1.167 (0.931–1.464)	0.180/0.720
rs28372683	CC	—	—	—	—
	CA/AA	**0.618 (0.461–0.827)**	**0.001**	**0.499 (0.366–0.681)**	**1.2e-5/4.8e-5**
**(b) SNPs associated with the spontaneous clearance of HCV infection, Group B** ***vs*** *.* **Group C**					
rs842647	GG	—	—	—	—
	GA/AA	1.045 (0.740–1.477)	0.802	1.041 (0.489–2.218)	0.917/3.668
rs7101916	CC	—	—	—	—
	CT/TT	0.918 (0.668–1.262)	0.599	0.650 (0.331–1.275)	0.210/0.840
rs12769316	CC	—	—	—	—
	CT/TT	1.100 (0.784–1.543)	0.580	0.995 (0.504–1.963)	0.988/3.952
rs28372683	CC	—	—	—	—
	CA/AA	0.928 (0.563–1.528)	0.736	0.683 (0.179–2.599)	0.576/2.304

Group A: controls; Group B: spontaneous clearance subjects; Group C: persistent infection patients.

Group (B + C): Infected individuals, including persistent infection group and spontaneous clearance group.

^a^Unadjusted *P* value, odds ratio (OR) and 95% confidence intervals (CI).

^b^The *P* value, odds ratio (OR), 95% confidence intervals (CI) were calculated on the basis of the binary logistic regression model, adjusted by gender, age, route of infection (and HCV genotype) in dominant model (GG vs. GA + AA for rs842647, CC vs. CT + TT for rs7101916, CC vs. CT + TT for rs12769316, CC vs. CA + AA for rs28372683).

^c^Multiple testing: using Bonferroni correction.

Further stratification analysis (Table 4) indicated that, compared with the major allele homozygote (rs7101916 CC genotype or rs28372683 CC genotype), a significant decreased risk was found in rs7101916 T allele or rs28372683A allele in the male subgroup (OR = 0.734, 95% CI = 0.572–0.941, *P* = 0.015) for rs7101916 and in all subgroup for rs28372683 (all *P* < 0.05, shown in Table 4), after adjusting for gender, age, and the route of infection.

**Table 4:** Stratified analysis of rs7101916 and rs28372683 among control, spontaneous clearance and persistent infection groups.

SNPs	Allele	Subgroups	Group (B + C)/Group A		Group C/Group B	
			OR (95% CI)^a^	*P* ^a^	OR (95% CI)^b^	*P* ^b^
rs7101916	C/T	Age				
		<55	0.891 (0.705–1.125)	0.331	1.047 (0.727–1.508)	0.805
		≥55	**0.748 (0.611–0.915)**	**0.005**	0.860 (0.622–1.187)	0.359
		Gender				
		male	**0.734 (0.572–0.941)**	**0.015**	1.125 (0.731–1.731)	0.593
		female	0.842 (0.694–1.021)	0.080	0.812 (0.606–1.088)	0.163
		Routes of infection				
		HD	0.788 (0.599–1.037)	0.089	0.925 (0.573–1.494)	0.751
		Blood donation	1.002 (0.746–1.344)	0.992	0.878 (0.666–1.159)	0.359
rs28372683	C/A	Age				
		<55	**0.563 (0.368–0.862)**	**0.008**	0.623 (0.316–1.226)	0.171
		≥55	**0.589 (0.402–0.862)**	**0.006**	0.744 (0.416–1.330)	0.318
		Gender				
		male	**0.477 (0.294–0.774)**	**0.003**	0.780 (0.328–1.859)	0.576
		female	**0.640 (0.452–0.906)**	**0.012**	0.642 (0.384–1.074)	0.092
		Routes of infection				
		HD	**0.394 (0.196–0.795)**	**0.009**	0.596 (0.140–2.532)	0.483
		Blood donation	**0.632 (0.461–0.867)**	**0.004**	0.685 (0.431–1.091)	0.111

Abbreviations: CI, confidence interval; HD, hemodialysis; OR, odds ratio.

Group A: controls; Group B: spontaneous clearance subjects; Group C: persistent infection patients. Group (B + C): HCV-infected individuals.

^a^The *P* value, OR and 95% CIs of group (B + C) versus Group A were calculated on the basis of the binary logistic regression model, adjusted by gender, age and routes of infection in dominant model (CC vs. CT + TT for rs7101916, CC vs. CA + AA for rs28372683).

^b^The *P* value, OR and 95% CIs of group C versus Group B were calculated on the basis of the binary logistic regression model, adjusted by gender, age, routes of infection and HCV genotype in dominant model (CC vs. CT + TT for rs7101916, CC vs. CA + AA for rs28372683).

Bold type indicates statistically significant results.

### Association between *NF-κB* polymorphisms and spontaneous clearance of HCV infection

No association was found between the four SNPs (rs842647, rs7101916, rs12769316 and rs28372683) and spontaneous clearance of HCV in our logistic regression analysis using dominant model, adjusting for gender, age, the route of infection and HCV genotype (all *P* > 0.05, Table 3), or in the further stratification analysis (all *P* > 0.05, Table 4).

### Cumulative effects of rs7101916 and rs28372683 on the susceptibility of HCV infection

The analysis of combined protective alleles (rs7101916 T allele and rs28372683 A allele) suggested that subjects carried 1–3 protective alleles was associated with a decreased risk of the susceptibility of HCV infection when compared with subjects carried 0 protective allele (all *P* < 0.05, Table 5). Additionally, these two SNPs have trended influence on a decreased risk of the susceptibility of HCV infection after the Cochran–Armitage trend test (OR = 0.641, 95% CI = 0.514–0.799, *P* = 7.9e-5, Table 5). Moreover, after analyzing the combined protective genotypes (rs7101916 TT and rs28372683 AA) on the susceptibility to HCV infection shown in Table 6, we found that carrying 1 protective genotypes offered a significant high protective effect (OR = 0.696, 95% CI = 0.492–0.984, *P* = 0.04).

**Table 5:** Cumulative effects of combined risk alleles (rs7101916 T and rs28372683 A) on the susceptibility to HCV infection.

Variables	Group A	Group (B + C)	Group (B + C)/Group A	
	n	n	OR(95% CI)^a^	*P* ^a^
0	302	277	1	
1	439	296	**0.702 (0.554–0.890)**	**0.003**
2	189	94	**0.543 (0.396–0.747)**	**1.68e-4**
3	31	8	**0.254 (0.111–0.580)**	**0.001**
4	1	4	3.974 (0.403–39.226)	0.237
Trend				**8e-6** ^**b**^
1–4	660	402	**0.641 (0.514–0.799)**	**7.9e-5**

Abbreviations: HCV, hepatitis C virus; OR, odds ratio; 95% CI, 95% confidence interval.Group A: controls; Group B: spontaneous clearance subjects; Group C: persistent infection patients. Group (B+C): HCV-infected individuals. ^a^Logistic regression model, adjusted for gender, age and routes of infection. ^b^Cochran–Armitage trend test. Bold type indicates statistically significant results.

**Table 6:** Cumulative effects of combined risk genotypes (rs7101916 TT and rs28372683 AA) on the susceptibility to HCV infection.

Variables	Group A	Group (B + C)	Group (B + C)/Group A	
	n	n	OR (95% CI)^a^	*P* ^a^
0	302	277	1	
1	127	80	**0.696 (0.492–0.984)**	**0.040**
2	1	4	4.112 (0.407–41.595)	0.231
Trend				0.087^b^
1–2	128	84	0.725 (0.515–1.021)	0.065

Abbreviations: HCV, hepatitis C virus; OR, odds ratio; 95% CI, 95% confidence interval.

Group A: controls; Group B: spontaneous clearance subjects; Group C: persistent infection patients. Group (B + C): HCV-infected individuals.

^a^Logistic regression model, adjusted for gender, age and routes of infection.

^b^Cochran–Armitage trend test.

Bold type indicates statistically significant results.

### Haplotype analysis of rs7101916 and rs28372683 on the susceptibility of HCV infection

The two-locus haplotypes were consisted of rs7101916 and rs28372683 variant alleles. Compared with the most frequent CC haplotype, TC and CA haplotype were significantly associated with the decreased risk of the susceptibility to HCV infection (TC haplotype: OR = 0.68, 95% CI = 0.568–0.813, *P* < 0.001; CA haplotype: OR = 0.604, 95% CI = 0.425–0.858, *P* = 0.005) (Table 7).

**Table 7:** Haplotype frequencies of rs7101916 and rs28372683.

Variables	Group A	Group (B + C)	Group (B + C)/Group A	
	n	n	OR (95% CI)^a^	*P* ^a^
CC	1045	850	1	
TC	709	424	**0.680 (0.568–0.813)**	**<0.001**
CA	139	68	**0.604 (0.425–0.858)**	**0.005**
TA	33	16	0.557 (0.280–1.109)	0.086

Abbreviations: HCV, hepatitis C virus; OR, odds ratio; 95% CI, 95% confidence interval.

Group A: controls; Group B: spontaneous clearance subjects; Group C: persistent infection patients. Group (B + C): HCV-infected individuals.

^a^Logistic regression model, adjusted for gender, age and routes of infection.

Bold type indicates statistically significant results.

### *In silico* analysis of SNPs function

**Figure 2:**
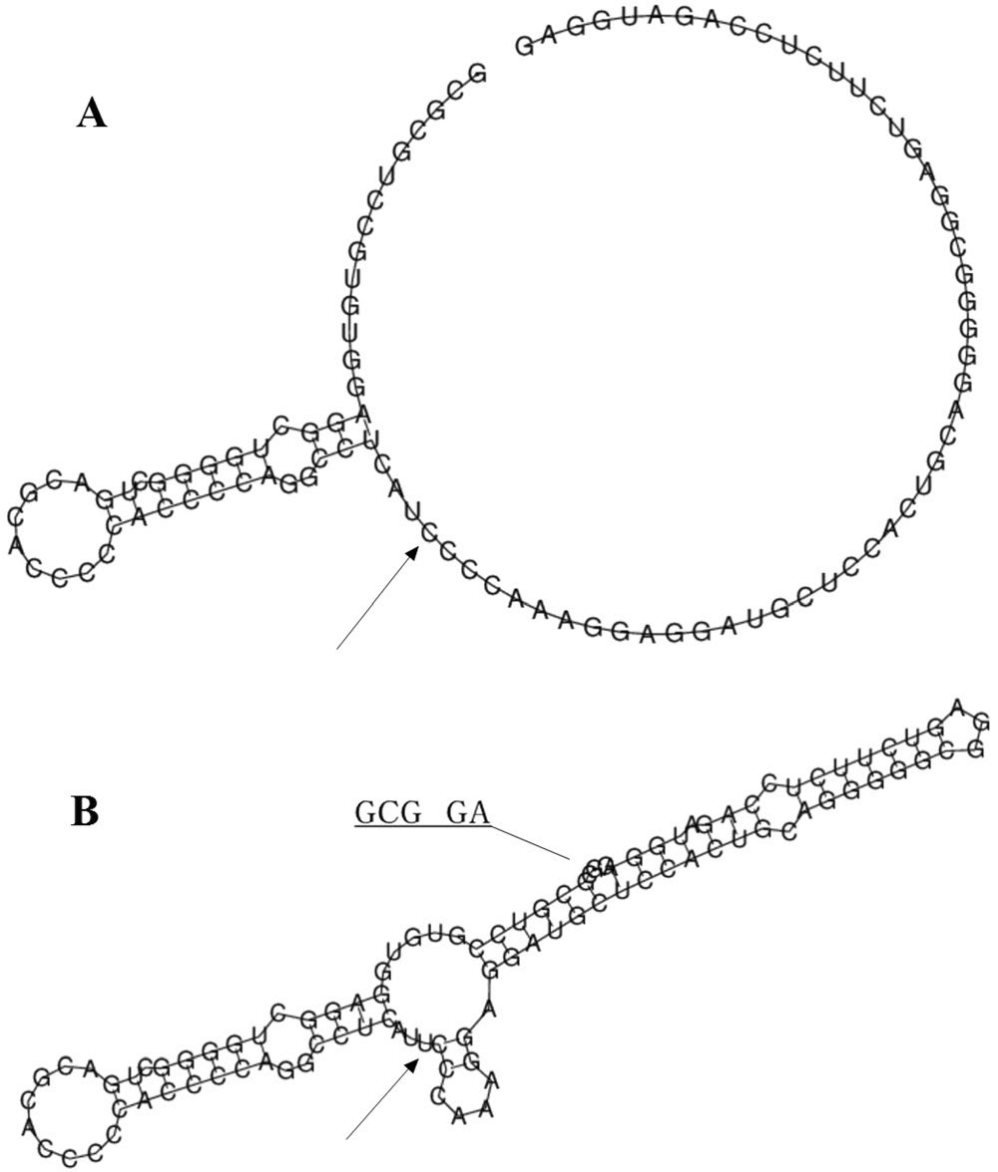
The influence of rs7101916 on mRNA centroid secondary structures of RelA near the 5′end region. Changes in the local structure were illustrated by the RNAfold Web Server. The arrow indicates the position of the mutation (50 bases upstream and 50 bases downstream from the mutation). The minimum free energy of the mRNA centroid secondary structure (a structure with minimal base pair distance) for wild type and mutant rs7101916 were estimated to be −44.70 kcal/mol (Fig. 2A) and −49.90 kcal/mol (Fig. 2B), respectively. The wild-type and mutant-type sequences are listed below. Underline type indicates the overlapping nucleotide letter that are unreadable in Fig. 2B. The underline bold type indicates the nucleotide difference between the wild and mutant allele. Wild-type sequence: GCGCGUCCGUGUGGAGGCUGGGGCUGACGCACCCCCACCCCAGGCCUCAU**C**CCCAAAGGAGGAUGCUCCACUGCAGGGGGCGGAGUCUUCUCCAGAUGGAG Mutant-type sequence: GCGCGUCCGUGUGGAGGCUGGGGCUGACGCACCCCCACCCCAGGCCUCAU**U**CCCAAAGGAGGAUGCUCCACUGCAGGGGGCGGAGUCUUCUCCAGAUGGAG.

Rs7101916 is located near the 5′ end of the RelA gene, which contains 11 exons and is mapped to chromosome 11q13.1. Using the SNP Information file (SNPInfo) web server, rs7101916 was predicted to be a transcription factor binding site (TFBS). Combined with the location of rs7101916, this mutation could involve in altering the binding of TF and mediating the transcriptional regulation. Therefore, RelA RNA secondary structures with rs7101916 major allele or minor allele were further predicted through energy minimization using RNAfold web server, based on the latest ViennaRNA Package (Version 2.4.6). The local structure changes are shown in Fig. 2. The minimum free energy of the centroid secondary structure (a structure with minimal base pair distance) for minor T allele of rs7101916 (−49.90 kcal/mol) was lower than that of major C allele (−44.70 kcal/mol). Results of eQTL analysis indicated that different genotypes of rs7101916 could cause differential mRNA level of ribonuclease H2 subunit C (RNASEH2C) in transformed fibroblasts cells and influence liver fibrosis process after HCV infection.

**Figure 3:**
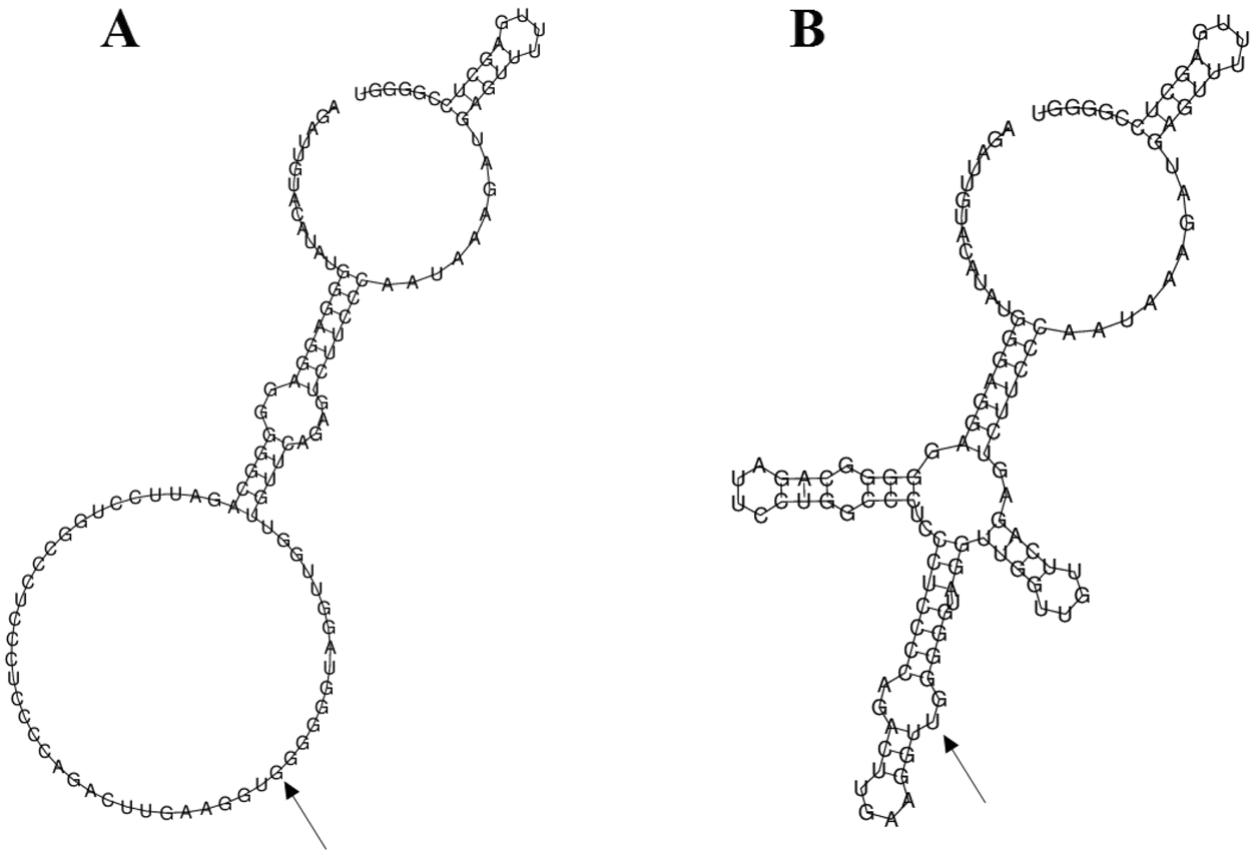
The influence of rs28372683 on mRNA centroid secondary structures of RelB in the 3′-UTR region. Changes in the local structure were illustrated by the RNAfold Web Server. The arrow indicates the position of the mutation (60 bases upstream and 60 bases downstream from the mutation). The minimum free energy of the mRNA centroid secondary structure (a structure with minimal base pair distance) for wild type and mutant rs28372683 were estimated to be −24.30 kcal/mol (Fig. 3A) and −35.00 kcal/mol (Fig. 3B), respectively. The wild-type and mutant-type sequences are listed below. The underline bold type indicates the nucleotide difference between the wild and mutant allele. Wild-type sequence: AGAUUGUACAUAUGGGAGGAGGGGGCAGAUUCCUGGCCCUCCCUCCCCAGACUUGAAGGU**G**GGGGGUAGGUUGGUUGUUCAGAGUCUUCCCAAUAAAGAUGAGUUUUUGAGCCUCCGGGGU Mutant-type sequence: AGAUUGUACAUAUGGGAGGAGGGGGCAGAUUCCUGGCCCUCCCUCCCCAGACUUGAAGGU**U**GGGGGUAGGUUGGUUGUUCAGAGUCUUCCCAAUAAAGAUGAGUUUUUGAGCCUCCGGGGU.

Rs28372683 is located in the 3′-untranslated region (3′-UTR) region of RelB gene, which contains 12 exons and is mapped to chromosome 19q13.32. According to the SNPinfo web server, rs28372683 was predicted to be a TFBS or a micro RNA (miRNA) (hsa-miR-1224-3p, hsa-miR-532-3p) binding site, or involved in the exotic splicing (enhancer or silencer). Considering the possible RelB gene expression regulation effect of rs28372683 variation at the translational or post-translational level, RelB RNA secondary structure was further predicted using RNAfold web server. The local structure changes are shown in Fig. 3. The minimum free energy of the centroid secondary structure for mutant A allele (corresponded to U allele in Fig. 3) of rs28372683 (−35.00 kcal/mol) was lower than that of wild C allele (corresponded to G allele in Fig. 3, −24.30 kcal/mol).

Rs12769316 is located near the 5′ end of the NF-κB2 gene, which contains 25 exons and is mapped to chromosome 10q24.32. Using the SNPinfo web server, rs12769316 was predicted to be a TFBS and could be involved in transcriptional regulation. Then we also analyzed NF-κB2 RNA secondary structure using RNAfold web server. However, no difference between wild type and mutant type was found (Supplementary Fig. 1). The lowest free energy of the centroid secondary structure for the wild C allele (corresponded to G allele in Supplementary Fig. 1, −23.40 kcal/mol) of rs12769316 is nearly identical to that of the mutant T allele (corresponded to A allele in Supplementary Fig. 1, −24.40 kcal/mol). Therefore, rs12769316 could not influence the transcriptional control of NF-κB2 gene expressions or HCV infection.

## Discussion

Our study firstly indicated that RelA rs7101916 T allele and RelB rs28372683 A allele were associated with the decreased risk of the susceptibility to HCV infection among the Chinese Han population. Host genetic background strongly influences the susceptibility and the response to HCV infection. Our previous studies, as well as other previous studies, have showed that many genetic variants affect HCV infection immune response and related to different disease outcomes, such as RelA^19^, Toll-like receptor 7^31,32^, interleukin-18^33^, human leukocyte antigen class II^33–36^, vitamin D receptor^37,38^ and estrogen receptor α^39^.

In this study, RelA rs7101916 (located near the 5′ end of the gene) mutant T allele was found to be linked to the protection from HCV infection. RelA gene encoded transcription factor p65, also known as NF-κB p65 subunit, which formed the heterodimeric p65-p50 complex, the most abundant one of the transamination complexes^11^. According to the *in silico* analysis in this study, the minimum free energy of the local structure for T allele of rs7101916 was lower than that of C allele. Considering that rs7101916 located near the 5′ end of the RelA gene, it may be a TFBS and influence the binding of TF (according to the SNPInfo), followed by the transcriptional regulation^40,41^. Besides, according to the information from UCSC (http://www.genome.ucsc.edu) and the results of eQTL analysis, this SNP located at regulatory elements region, can have possible functions on gene transcription, expression process and influence liver fibrosis process after HCV infection excepting the association with HCV susceptibility. Therefore, the genetic variation of rs7101916 may influence the RelA gene transcriptional regulation and gene expression, subsequently affecting NF-κB pathway activation and the susceptibility to HCV infection.

In our study, RelB rs28372683 (located in the 3′-UTR region of the gene) mutant A allele was associated with the protection from HCV infection. RelB is an important arm of the RelB/p52 NF-κB complex in a non-canonical NF-κB pathway activation through a mechanism dependent on inducible processing of p100^11,15^. According to the results of *in silico* analysis, rs28372683 location could not only bind to the transcription or miRNAs, but also be exotic splicing (enhancer or silencer) site. In addition, the minimum free energy of the centroid secondary structure for A allele of rs28372683 was lower than that of C allele. Therefore, the genetic variation of RelB rs28372683 may be associated NF-κB pathway activation and the susceptibility to HCV infection through potential functional mechanisms.

The results of the cumulative effects and haplotype analyses suggested that the more protective alleles (rs7101916 T and rs28372683 A) subjects carried, the greater the protective effect on the susceptibility of HCV infection exhibited, except that some groups with negative results had too few subjects to detect with sufficient statistical power.

There were no significant relationship of rs842647 and rs12769316 to spontaneous clearance of HCV infection. Rs842647 is located in the intron region of RelB gene. Introns on DNA are transcribed into the precursor RNA, the introns on the RNA are cleaved before the RNA leaves the nucleus for translation, and ultimately not in the mature RNA molecule. Rs12769316 is located 1.5 kb upstream of 5′ of the NF-κB2 gene and was found to be related to NF-κB2 protein and mRNA expression and predicted to be a TFBS. However, we did not find the correlation between rs12769316 and HCV clearance. The reason may be that the SNP is meaningless in our ethnicity and the number of subjects included in the study is insufficient. More studies should be performed to confirm this result.

Several limitations of our study merit consideration. First, it is difficult to acquire the subjects’ exact age and time of the initial infection with HCV, thereby possibly impacting subjects’ immune response and outcomes of HCV infection. Second, the partial lack of HCV genotypes among spontaneous clearance subjects and persistent infection patients could lead to unreliable analysis of the association between NF-κB polymorphisms and spontaneous clearance of HCV infection, especially for the compilation of the genotypes distributions between spontaneous clearance subjects v.s. persistent infection patients. Therefore, more studies were needed to confirm the negative results of the associations between the four SNPs and spontaneous clearance of HCV infection in this study. Direct-acting antiviral (DAA) regimens have reformed the treatment of HCV. Predicting treatment response to an IFN-based regimen is still far from enough. However, the new therapy has not been used extensively because of its unknown adverse effects and expensive costs in developing countries like China. As it was before, PEG-IFN/RBV regimen is still the first-line treatment for patients with HCV type 1 infection in China. Future studies should focus on the relationship between genetic polymorphisms and DAA treatment response.

In conclusion, this study firstly reported that the carriage of RelA rs7101916 T or RelB rs28372683 A was the potential protective factor against HCV infection among the Chinese population. These findings can serve as a reference for the preventive, predictive and therapeutic strategies of HCV infection. Additionally, further epidemiological and functional research on the *NFKB* pathway genetic variants is required.

## Supplementary information

Supplementary Information
