# Supplementary material for: Genetic mutations in NF-κB pathway genes were associated with the protection from hepatitis C virus infection among Chinese Han population
Source: Sci Rep. 2019 Jul 25;9:10830. doi: 10.1038/s41598-019-47058-y (PMC6658546; doi:10.1038/s41598-019-47058-y)
Supplement: Supplementary file 1 — Supplementary Information [file 41598_2019_47058_MOESM1_ESM.doc]

**Genetic mutations in NF-κB pathway genes were associated with the protection from hepatitis C virus infection among Chinese Han population**

Ming Yue^1^, Ting Tian^2^, Chunhui Wang^3^, Haozhi Fan^4^, Jingjing Wu^4^, Jinke Wang^5^, Jun Li^1^, Xueshan Xia^6^, Amei Zhang^6^, Rongbin Yu^4^, Yun Zhang^3, 4^, Peng Huang^4*^

1 Department of Infectious Diseases, The First Affiliated Hospital of Nanjing Medical University, Nanjing 210029, China;

2 Department of Acute Infectious Disease Control and Prevention, Jiangsu Province Center for Disease Prevention and Control, Nanjing 210009, China;

3 Institute of Epidemiology and Microbiology, Eastern Theater Command Centers for Disease Control and Prevention, Nanjing 210002, China;

4 Department of Epidemiology and Biostatistics, Key Laboratory of Infectious Diseases, School of Public Health, Nanjing Medical University, Nanjing 211166, China;

5 State Key Laboratory of Bioelectronics, Southeast University, Nanjing 210096, China;

6 Faculty of Life Science and Technology, Kunming University of Science and Technology, Kunming 650500, China

Correspondence and requests for materials should be addressed to P. H. (E-mail: [huangpeng@njmu.edu.cn](mailto:huangpeng@njmu.edu.cn), Tel/Fax: 86 25 86868291)

**Keywords:** NF-κB, genetic polymorphism, HCV, infection, susceptibility

**Fund programs:** Grants from the Natural Science Foundation of Jiangsu Province of China (grant numbers: BK20171054, BK20151026); the National Natural Science Foundation of China (grant numbers: 81703273, 81502853, 81773499, 61571119); Jiangsu Program for Young Medical Talents (grant number: QNRC2016616); and the Medical Innovation Team Project of Jiangsu Province (grant number: CXTDA2017023); Priority Academic Program Development of Jiangsu Higher Education Institutions (PAPD).

**Supplementary Table 1 Probes and primers of investigated *NF-κB* SNPs for TaqMan assay.**

| SNPs (genotype) | Gene | Region | MAF^a/b^ | TaqMan-MGB probe/primers sequences |
| --- | --- | --- | --- | --- |
| rs842647 G>A | Rel | Intron | 0.159/0.183 | Probe-G: FAM-CCACTTTATAAATGCAGTTT-MGB |
|  |  |  |  | Probe-A: HEX-AAATACCACTTTATAAATACA-MGB |
|  |  |  |  | Forward primer: TGATTCTTGGGTCATTGACTGA |
|  |  |  |  | Reverse primer: TGGGCGACAAGTGTGAAACTC |
| rs7101916 C>T | RelA | nearGene-5 | 0.385/0.427 | Probe-C: FAM-AGGCCTCATCCCCAA -MGB |
|  |  |  |  | Probe-T: HEX-AGGCCTCATTCCCAA -MGB |
|  |  |  |  | Forward primer: GGTAGCTCCCCCAAGACCAA |
|  |  |  |  | Reverse primer: AATCTTTTCCAATTCCCACATGA |
| rs12769316 C>T | NF-κB2 | nearGene-5 | 0.164/0.133 | Probe-C: FAM-CCCAGACGTTTTTAA-MGB |
|  |  |  |  | Probe-T: HEX-CCCCAGATGTTTTT-MGB |
|  |  |  |  | Forward primer: CGGTCTTTCCTGCAGCTT |
|  |  |  |  | Reverse primer: CCAAATCGAGAGCCATAATAGTCTCT |
| rs28372683 C>A | RelB | 3’-UTR | 0.089/0.100 | Probe-C: FAM-CCTACCCCCCACCTT-MGB |
|  |  |  |  | Probe-A: HEX-TACCCCCAACCTTCAA-MGB |
|  |  |  |  | Forward primer: TTGGGAAGACTCTGAACAACCA |
|  |  |  |  | Reverse primer: CAGTAGGATTCGGAAAAGATTGTACA |

MAF: minor allele frequency.

a minor allele frequencies in control group.

b minor allele frequencies from HapMap of Han Chinese in Beijing, China (CHB) (dbSNP, build128; available at http://www.ncbi.nlm.nih.gov/SNP/).


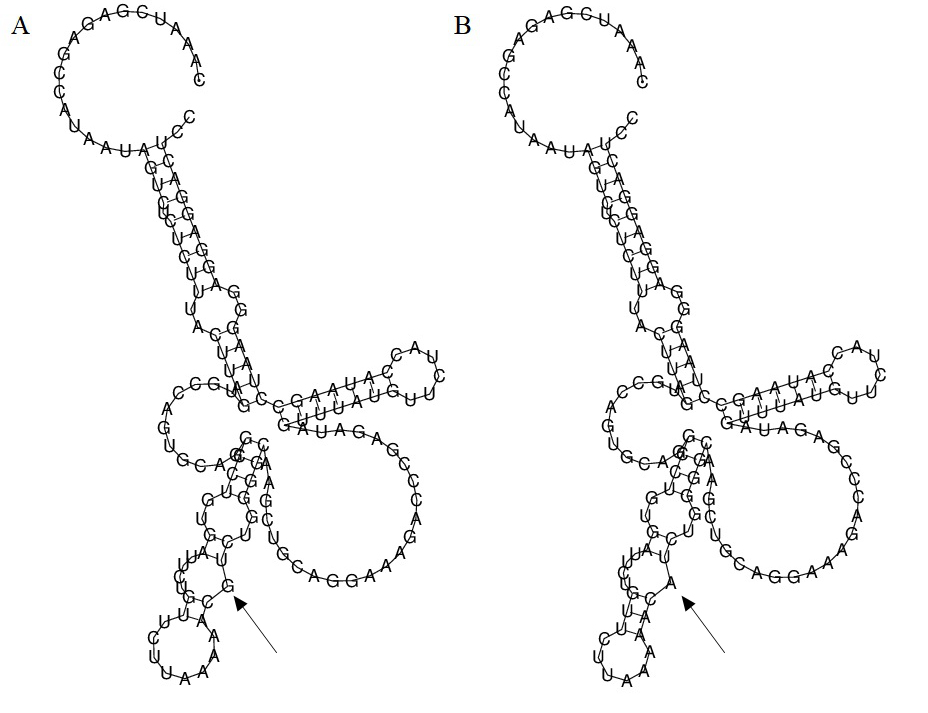


Supplementary Figure 1. The influence of rs12769316 on mRNA centroid secondary structures of NF-κB2 near the 5’end region. Changes in the local structure were illustrated by the RNAfold Web Server. The arrow indicates the position of the mutation (70 bases upstream and 70 bases downstream from the mutation). The minimum free energy of the mRNA centroid secondary structure (a structure with minimal base pair distance) for wild type and mutant rs12769316 were estimated to be −23.40 kcal/mol (Supplementary Figure 1A) and −24.40 kcal/mol (Supplementary Figure 1B), respectively. The underline bold type indicates the nucleotide difference between the wild and mutant allele.

Wild-type sequence:

CAAAUCGAGAGCCAUAAUAGUCUCUCUUUACUUAGUGCCAGUGCAGGCCUGUGAUUCUGUUCUUAAAAAC**G**UCUGGGGCAAGCUGCAGGAAAGACCCGAGAUAGUUUAUGUUCUACCAUAAGCCUAAGGGAGGAGGACUCC

Mutant-type sequence:

CAAAUCGAGAGCCAUAAUAGUCUCUCUUUACUUAGUGCCAGUGCAGGCCUGUGAUUCUGUUCUUAAAAAC**A**UCUGGGGCAAGCUGCAGGAAAGACCCGAGAUAGUUUAUGUUCUACCAUAAGCCUAAGGGAGGAGGACUCC
